# A Comparative Analysis of the Clinical Application of a Novel *Helicobacter pylori* Serum Antibody Typing Test and the ^13^C-Urea Breath Test

**DOI:** 10.3390/diagnostics15070934

**Published:** 2025-04-05

**Authors:** Chonghui Hu, Zhipeng Zhao, Dong Zhu, Runqing Li, Xuan Jiang, Yutang Ren, Xin Ma, Xiuying Zhao

**Affiliations:** 1 Department of Laboratory Medicine, Beijing Tsinghua Changgung Hospital, School of Clinical Medicine, Tsinghua Medicine, Tsinghua University, Beijing 102218, China; hchs00721@btch.edu.cn (C.H.); zzpa00111@btch.edu.cn (Z.Z.); zda00415@btch.edu.cn (D.Z.); lrqa00335@btch.edu.cn (R.L.); maxin9099@163.com (X.M.); 2 Department of Gastroenterology, Beijing Tsinghua Changgung Hospital, School of Clinical Medicine, Tsinghua Medicine, Tsinghua University, Beijing 102218, China; jxa01998@btch.edu.cn (X.J.); ryta01044@btch.edu.cn (Y.R.); 3 Department of Gastroenterology, Affiliated Hospital of Xuzhou Medical University, Xuzhou 221000, China

**Keywords:** *Helicobacter pylori*, antibody typing test, ^13^C-urea breath test, delta over baseline

## Abstract

**Background/Objectives**: To compare and analyze the application of a *Helicobacter pylori* (*H. pylori*, Hp) serum antibody typing test (Hp-sATT) and the ^13^C-urea breath test (^13^C-UBT) in the diagnosis of Hp infection against an empirical therapy background. **Methods**: The detection of Hp-sATT using a combination of the quantum dot immunofluorescence method and the ^13^C-UBT was carried out in 237 patients who visited the Department of Gastroenterology at Beijing Tsinghua Changgung Hospital. The diagnostic consistency and correlation with gastric lesions of the two detection methods were analyzed by integrating the detection results, clinical information, and special staining of Hp in histopathological tissues (SS-Hp). **Results**: For the ^13^C-UBT, 104 (43.88%) cases were positive and 133 (56.12%) were negative. Positive results were found in 127 (53.59%) patients by using the Hp-sATT, with 67 (28.27%) cases of Type I Hp infection and 60 (25.32%) cases of Type II Hp infection. The consistency analysis between the Hp-sATT and ^13^C-UBT for all the patients showed a Kappa value of 0.339 (*p* < 0.001); the consistency analysis between the Hp-sATT and the 127 patients with SS-Hp showed a Kappa value of 0.427 (*p* < 0.001); and the consistency analysis between the ^13^C-UBT and the 127 patients with SS-Hp indicated a Kappa value of 0.621 (*p* < 0.001). However, in 191 patients without a history of Hp eradication, the consistency analysis results for the three methods improved, with Kappa values of 0.467 (*p* < 0.001) and 0.457 (*p* < 0.001) for the Hp-sATT with the ^13^C-UBT and SS-Hp, respectively, and 0.646 (*p* < 0.001) for the ^13^C-UBT with SS-Hp. In addition, a positive correlation was found between the signal values of anti-urease antibodies and the Delta Over Baseline (DOB) values of the ^13^C-UBT. The results also indicated that Hp-infected patients exhibited more pronounced gastric lesions, while cases with Type I Hp infection did not. **Conclusions**: In patients without a history of Hp eradication, the consistency between the Hp-sATT and ^13^C-UBT is moderate. However, Hp eradication therapy can reduce the consistency of the test results. When screening for Hp infection using the Hp-sATT, it is necessary to consider the patient’s history of Hp eradication.

## 1. Introduction

*Helicobacter pylori* (*H. pylori*, Hp) is a Gram-negative spiral-shaped bacterium that can survive in the highly acidic environment of the human stomach. Globally, approximately more than 43.1% of the population are infected with this bacterium, yet there is a clear geographical difference in infection rates [1]. Hp infection is closely related to various gastric diseases, including chronic gastritis, peptic ulcers, mucosa-associated lymphoid tissue lymphoma, and gastric cancer [2]. The virulence factors of Hp, particularly the cytotoxin-associated protein A (CagA) and the vacuolating cytotoxin A (VacA), play a significant role in the severity of diseases following Hp infection. Studies have shown that CagA is the most important virulence factor of Hp, which can cause inflammation of the gastric mucosal tissue, leading to changes, proliferation, and atrophy of epithelial cells [3,4]. VacA has a direct toxic effect on gastric epithelial cells, causing vacuolation, necrosis, and ulceration of the epithelial cells, and can damage the gastric mucosa and delay the repair of the gastric epithelium [4]. Hp can be classified into Type I and Type II based on whether it expresses CagA and/or VacA [5]. If an Hp strain expresses either CagA or VacA, it is considered a Type I Hp infection; if neither CagA nor VacA is expressed, it is considered a Type II Hp infection. Previous studies have shown that compared to Type II Hp infection, Type I Hp infection can exacerbate gastric mucosal inflammation and significantly increase the risk of gastric cancer [6,7]. It is generally believed that after Hp infection, the immune cells of the infected individuals are activated to produce anti-urease antibodies, anti-CagA, and anti-VacA antibodies [8,9]. Clinically, by detecting the serum urease, CagA, and VacA antibodies in patients and combining the antibody repertoire to determine the type of Hp infection, the infection can be effectively assessed, providing important guidance for clinical management.

Conventional understanding suggests that the levels of specific antibodies in the blood of Hp-infected individuals are relatively stable and are not affected by the degree of infection or treatment [8]. As a result, the Hp serum antibody typing test (Hp-sATT) is more frequently used in check-ups in health centers or for epidemiological survey purposes. The urea breath test (UBT) utilizes the urease produced by Hp to break down orally administered labeled urea into ammonia and carbon dioxide. The presence of infection can be determined by detecting changes in the concentration of labeled carbon dioxide in the exhaled breath [10]. It has been recommended by multiple guidelines as the preferred method for detecting current Hp infection [11,12,13]. The 13C-urea breath test (13C-UBT) is universally recognized for its better safety and wider applicability. Currently, both the Hp-sATT and 13C-UBT are widely used in clinical practice [14]. However, with the increasing awareness of Hp infection in public society and the growing number of patients adopt empirical Hp eradication therapy, there are widespread conflicting results regarding the application of the Hp-sATT and 13C-UBT [14,15,16]. In addition, paradoxical results regarding the correlation between Hp antibody typing and gastric diseases has been reported [17,18]. Therefore, it is necessary to conduct a comprehensive analysis of the clinical application of the Hp-sATT and 13C-UBT against the background of Hp eradication therapy.

This study is a retrospective cohort analysis. Patients who underwent a Hp-sATT using semi-quantitative quantum dot immunofluorescence were included; by integrating the results of their ^13^C-UBT, previous treatments, and special staining of Hp in histopathological tissues (SS-Hp), the study aimed to elucidate the role of the Hp-sATT, as compared with the ^13^C-UBT, in the clinical diagnosis of Hp-related diseases.

## 2. Materials and Methods

### 2.1. Subject Enrollment

This study included 237 patients who visited the Department of Gastroenterology at Beijing Tsinghua Changgung Hospital between September 2023 and September 2024, presenting with chronic gastrointestinal symptoms. Patients with major systemic comorbidities or malignancies were excluded to minimize confounding effects. Repeated visits were also excluded. To ensure the validity of test results, all participants were required to discontinue antibiotics, proton pump inhibitors, bismuth-containing medications, and other potentially interfering medications for at least 14 days prior to testing. The average age of the 237 patients was 46.90 ± 13.89 years. All of the 237 patients underwent a ^13^C-UBT and Hp-sATT, and clinical data were collected anonymously. A total of 127 patients undergoing gastric mucosal pathology and SS-Hp were divided into four groups based on the results of gastroscopy and/or gastric mucosal pathology: the chronic non-atrophic gastritis (gastritis) group, the chronic non-atrophic gastritis with erosion (erosion) group, the gastric or duodenal ulcer (ulcer) group, and the chronic atrophic gastritis, intestinal metaplasia, or dysplasia (atrophy/intestinal metaplasia/dysplasia) group. The grouping was based on the most severe lesion found in the diagnostic results that were available for review.

### 2.2. Detection Methods and Criteria

The Hp-sATT was performed using the Hp typing test kit (semi-quantitative quantum dot immunofluorescence method, Chongqing Xinsaiya Biotechnology Co., Ltd., Chongqing, China), which can read the signal values of each item. The following interpretation criteria were established, according to the instructions: anti-urease antibody detection ≥ 8 RU/mL is positive, CagA antibody detection ≥ 6 RU/mL is positive, and VacA antibody detection ≥ 4 RU/mL is positive. If all urease, CagA, and VacA antibodies are negative, the test is considered negative. If the anti-urease antibody is positive and either the CagA or VacA antibody is positive or both are positive, it is considered positive for Type I Hp infection. If the anti-urease antibody is positive and both the CagA and VacA antibodies are negative, it is considered positive for Type II Hp infection. The ^13^C-UBT was conducted using the HY-IREXB^13^C breath test instrument and its accompanying reagents (produced by Huayou Mingkang Optoelectronic Technology Co., Ltd., Guangzhou, China). A ^13^C-UBT DOB value ≥ 4 is considered positive, and a DOB value < 4 is considered negative. Gastroscopy was performed by senior-certified endoscopists using a Fujifilm EG-760Z video gastroscope (Fujifilm Corporation, Tokyo, Japan). Biopsies were obtained from gastric mucosa with visible lesions and processed for histopathological analysis. SS-Hp was manually stained with Baso *Helicobacter pylori* Stain (methylene blue method, Baso Diagnostics, China) and examined by two professional pathologists using an OLYMPUS BX53F microscope (Olympus Corporation, Japan) for the detection of Hp. A positive result was defined by the observation of typical spiral-shaped bacterial morphology, while the absence of such morphology was recorded as negative. The two professional pathologists examining the SS-Hp were blinded to the Hp-sATT and ^13^C-UBT results during their assessments to minimize observational bias.

### 2.3. Data Processing and Statistics

All statistical analyses were conducted using IBM SPSS Statistics version 26.0 (IBM Corp., Armonk, NY, USA). Continuous variables were described using the mean ± standard deviation (mean ± SD), and group comparisons were made using the Mann–Whitney U test. Correlation analysis was performed using the Spearman rank correlation test. Categorical data were described using frequencies and percentages (N, %), and group comparisons were made using the Chi-square test, Yates’ corrected Chi-square test, or Fisher’s exact probability test. Methodological comparisons were made based on the positive and negative agreement rates and consistency (Kappa > 0.60 indicates strong consistency; 0.40 < Kappa < 0.60 indicates moderate consistency; Kappa < 0.4 indicates poor consistency). All statistical tests were considered statistically significant if *p* < 0.05.

### 2.4. Ethics

Outpatient physicians determined whether to perform gastroscopy and histopathological examination based on the patient’s condition. Informed consent was obtained from all patients before the examination. The Hp-sATT was performed using residual samples from routine clinical testing, and exemption of informed consent was approved by the Institutional Review Board; the data were analyzed retrospectively, and did not involve patient privacy information (22569-2-01).

## 3. Results

### 3.1. General Information and Results

Among the 237 patients, there were 110 males and 127 females, with an average age of 46.90 ± 13.89 years. All 237 patients completed the Hp-sATT, of whom 127 (53.6%) had positive Hp antibodies and 110 (46.4%) had negative results. The number of patients with Type I Hp infection was 67 (28.27%), and with Type II Hp infection was 60 (25.32%). Among the 67 patients with Type I Hp infection, all were positive for the CagA antibody; 33 cases were positive for the VacA antibody, which was accompanied by the CagA antibody in all of these cases. Among the 237 patients, 104 (43.9%) had positive ^13^C-UBT results and 133 (56.1%) had negative results. Among the 127 patients who underwent gastric mucosal pathology and SS-Hp, 45 (35.4%) were positive and 82 (64.6%) were negative.

According to the “National Consensus on the Management of *Helicobacter pylori* Infection (Sixth Edition)” [13], patients with Hp positivity detected by the ^13^C-UBT and/or SS-Hp were considered to have active Hp infection. In this study, there were 107 (45.15%) patients with active Hp infection, including three patients who were ^13^C-UBT-negative but SS-Hp-positive, and 130 (54.5%) patients were classified as having a non-active Hp infection. No statistically significant differences were found in gender, age, and gastrointestinal symptoms between the two groups of patients (Table 1).

### 3.2. Comparison and Quantitative Analysis of Hp-sATT and ^13^C-UBT

A comparison and analysis of the Hp-sATT and ^13^C-UBT results from the 237 patients was conducted. The positive agreement rate between the two methods was 73.08% (76/104), and the negative agreement rate was 61.65% (82/133). The consistency analysis showed a Kappa = 0.339 (*p* < 0.001), indicating poor consistency between the two methods (Table 2).

Considering that the eradication of Hp might affect the test results, re-analysis was performed for 46 patients with a history of Hp eradication. The positive agreement rate between the two test methods was 55.56% (10/18), and the negative agreement rate was 28.57% (8/28). The consistency analysis showed a Kappa of −0.142 (*p* = 0.270), indicating consistency lower than the random expectation between the two methods (Table 2). Meanwhile, in the 191 patients without Hp eradication, the positive agreement rate between the two test methods was 82.50% (66/80), and the negative agreement rate was 70.48% (74/105). The consistency analysis showed a Kappa = 0.467 (*p* < 0.001), indicating moderate consistency between the two methods (Table 2). The results suggest that a history of Hp eradication was a key factor affecting the consistency of the results between the two methods. In patients with a history of eradication, the negative agreement of the two methods was significantly reduced.

**Figure 1:**
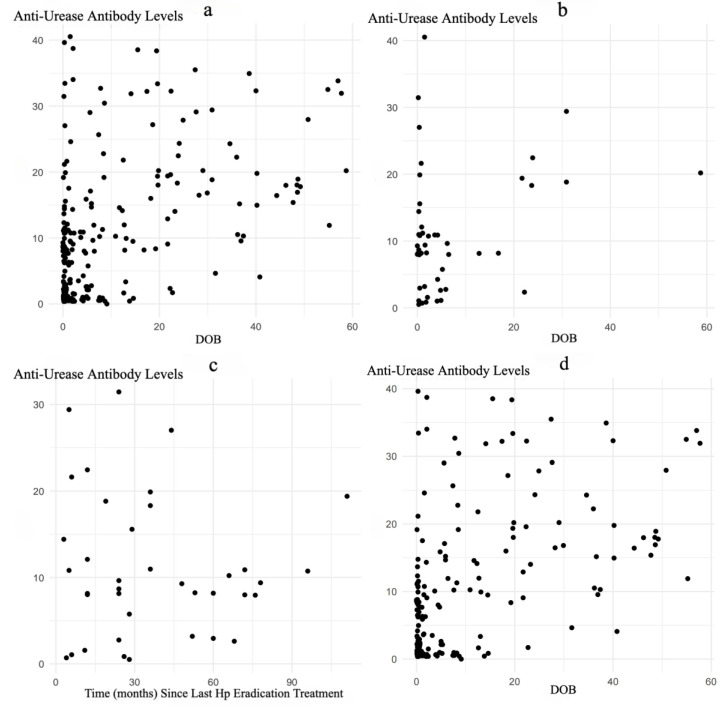
Comparison of anti-urease antibody levels and ^13^C-UBT DOB. Note: (**a**) Scatter plot of anti-urease antibody levels versus ^13^C-UBT DOB values. (**b**) Scatter plot of anti-urease antibody levels versus ^13^C-UBT DOB values in patients with history of Hp eradication treatment. (**c**) Comparison of anti-urease antibody levels with time (months) since last Hp eradication treatment. (**d**) Scatter plot of anti-urease antibody levels versus ^13^C-UBT DOB values in patients without history of Hp eradication treatment.

Given that the anti-urease antibody detection signal is a quantitative value, it is worth analyzing whether there is a correlation between this result and the ^13^C-UBT DOB result, as well as the impact of treatment interventions. A correlation analysis between the anti-urease antibodies of Hp and the ^13^C-UBT DOB indicated a correlation coefficient of *r* = 0.442 (*p* < 0.001), suggesting a positive correlation between the strength of the anti-urease antibodies and the ^13^C-UBT DOB result (Figure 1a). Further analysis of anti-urease antibody levels (*p* = 0.331) and ^13^C-UBT DOB values (*p* = 0.055) in relation to gastroscopy and/or histopathological diagnosis revealed no statistically significant differences (*p* = 0.331 and *p* = 0.055). A correlation analysis between the anti-urease antibodies and the ^13^C-UBT DOB in 46 patients with a history of Hp eradication showed a correlation coefficient of *r* = 0.066 (*p* = 0.663), which was not statistically significant (Figure 1b). The impact of the time since Hp eradication treatment was analyzed in 38 patients with a recorded time since the last Hp eradication, ranging from 3 to 111 months. The correlation coefficient between anti-urease antibody levels and time (months) since the last Hp eradication treatment was *r* = 0.154 (*p* = 0.355), which was not statistically significant (Figure 1c). A correlation analysis between the anti-urease antibodies and the ^13^C-UBT DOB in 191 patients without a history of Hp eradication showed a correlation coefficient of *r* = 0.489 (*p* < 0.001), which was statistically significant, suggesting a positive correlation between the strength of the anti-urease antibodies and the ^13^C-UBT DOB result (Figure 1d).

### 3.3. Comparison of Hp-sATT and ^13^C-UBT with SS-Hp

Among the 237 patients, 127 underwent SS-Hp. The comparison analysis between the Hp-sATT and SS-Hp showed a positive agreement rate of 88.89% (40/45) and a negative agreement rate of 59.76% (49/82); the consistency analysis showed a Kappa of 0.427 (*p* < 0.001), indicating moderate consistency between the two methods (Table 3). Among the 127 patients, 20 had a history of Hp eradication. The positive agreement rate between the two test methods was 100.00% (4/4), and the negative agreement rate was 50.00% (8/16); the consistency analysis showed a Kappa of 0.286 (*p* = 0.068). Among the 127 patients without Hp eradication, the positive agreement rate between the two test methods was 87.80% (36/41), and the negative agreement rate was 62.12% (41/66); the consistency analysis showed a Kappa of 0.457 (*p* < 0.001), indicating moderate consistency between the two methods (Table 3).

The comparison analysis between the ^13^C-UBT results and SS-Hp among the 127 patients showed a positive agreement rate of 93.33% (42/45) and a negative agreement rate of 74.34% (61/82); the consistency analysis showed a Kappa of 0.621 (*p* < 0.001), indicating moderate consistency between the two methods (Table 3). The positive agreement rate of the ^13^C-UBT was significantly better than that of the Hp-sATT. Among the 127 patients with a history of Hp eradication, the positive agreement rate was 100.00% (4/4), and the negative agreement rate was 68.75% (11/16); the consistency analysis showed a Kappa of 0.468 (*p* = 0.013), indicating moderate consistency between the two methods. Among the 107 patients without Hp eradication, the positive agreement rate was 92.68% (38/41), and the negative agreement rate was 75.76% (50/66); the consistency analysis showed a Kappa of 0.646 (*p* < 0.001), indicating moderate consistency between the two methods (Table 3).

### 3.4. Analysis of Hp-sATT and ^13^C-UBT with Gastroscopy and/or Histopathological Diagnosis

**Figure 2:**
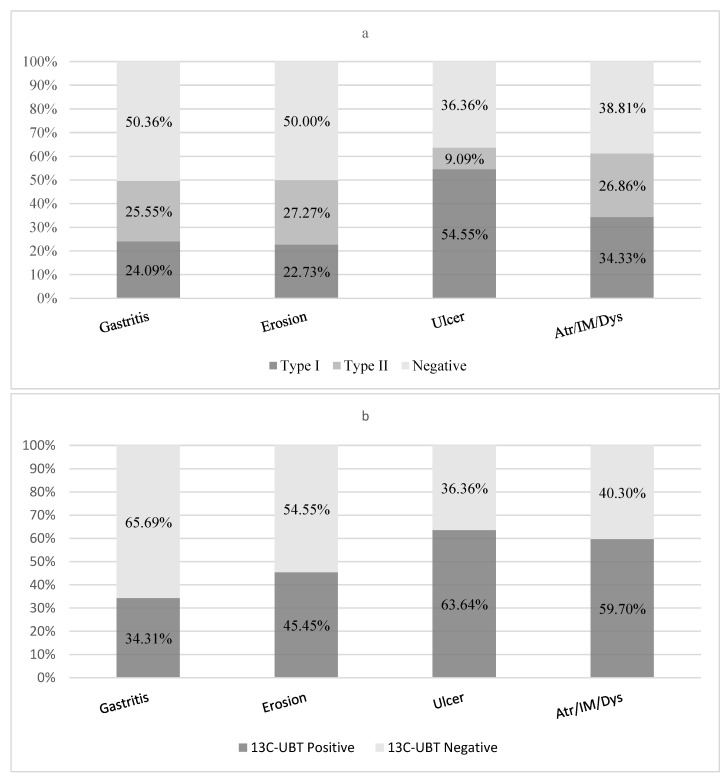
Hp-sATT and ^13^C-UBT with gastroscopy and/or histopathological diagnosis. Note: Atr/IM/Dys: atrophy/intestinal metaplasia/dysplasia. (**a**) Hp-sATT in relation to gastroscopic histopathological diagnostic categories. (**b**) ^13^C-UBT in relation to gastroscopic histopathological diagnostic categories.

A total of 237 patients with concurrent gastroscopy and/or histopathological diagnosis results were divided into four groups: gastritis, erosion, ulcer, and atrophy/intestinal metaplasia/dysplasia. The correlation between the results of the Hp-sATT and ^13^C-UBT and the histopathological changes was analyzed. Among the 237 patients, 67 (28.27%) were classified as Type I, 60 (25.32%) as Type II, and 110 (46.41%) were antibody-negative. No statistically significant correlation was found between the Hp-sATT and the disease groups (χ^2^ = 3.957, *p* = 0.266), nor was there a statistically significant difference in the positive rate of Hp antibodies between the disease groups (χ^2^ = 2.980, *p* = 0.395) (Table 4). However, the positive rate of Hp antibodies was higher in patients diagnosed with ulcers and atrophy/intestinal metaplasia/dysplasia compared to those with gastritis and erosion (Figure 2a).

Among the 237 patients, 104 (43.9%) were ^13^C-UBT positive, and 133 (56.1%) were negative. Similarly to the results of the Hp-sATT, the positive rate of the ^13^C-UBT was higher in patients diagnosed with ulcers and atrophy/intestinal metaplasia/dysplasia compared to those with gastritis and erosion (Figure 2b). There was a statistically significant difference in ^13^C-UBT results among the disease groups (χ^2^ = 13.675, *p* = 0.003) (Table 4).

## 4. Discussion

Hp infection affects half of the world’s population, making it a significant public health issue [19]. In China, the prevalence of Hp infection ranges from 36.2% to 86.8% [20], with notable regional differences. These differences may be related to local hygiene conditions, dietary habits, and the accessibility of medical resources. With the introduction of new guidelines [13,21,22], China has adopted a more proactive stance in recommending Hp eradication in the last two years. Non-invasive diagnostic methods, such as the Hp-sATT and the UBT, have been widely applied in clinical practice, due to their simplicity and efficiency [14]. However, each diagnostic method has its limitations. For example, the Hp-sATT requires several weeks for the body to develop specific antibodies after Hp infection, and the antibodies can be maintained in the serum for more than 6 months after the eradication of Hp [2]. Currently, the relationship between Hp infection and host immune response remains incompletely understood, so serological tests are not suitable for the early diagnosis of Hp infection and long term follow-up after treatment. Taking the UBT as an example, the results of the 13C-UBT may be affected by the patient’s dietary patterns and drug use, and there is a certain gray area [23]. Therefore, results from these two methods may not always be consistent, particularly in patients who have previously undergone Hp eradication therapy [24]. With the expansion of antibiotic therapy, the relationship between the results of the two commonly used clinical tests, the Hp-sATT and the 13C-UBT, and their clinical significance is worth further study. In this study, 237 patients with gastrointestinal symptoms were studied. Among them, 127 (53.59%) were positive for serum Hp antibodies, and 107 (45.15%) were confirmed to be infected with Hp through the 13C-UBT and/or SS-Hp, which is consistent with previous research results [20] and can provide basic data for epidemiological surveys in the Beijing area.

CagA and VacA are two important virulence factors of Hp, playing a key role in the histopathological process of Hp infection. Studies have shown that CagA can affect host cell signal transduction through different variations in its EPIYA motif, thereby promoting the occurrence of serious diseases such as gastric cancer [25]. VacA induces vacuolation and apoptosis of cells, further exacerbating the histopathological effects of Hp infection [26]. One study showed that the CagA gene was detected in 86 Hp-positive samples from patients with chronic gastritis, with a positive rate of 91.45% [27]. Another study showed that the CagA gene was widely present in Hp-infected individuals in China, especially in cases related to gastric cancer, with a higher positive rate [28]. Regarding the VacA gene, there are reports that its positivity rate in Hp-positive samples is almost 100% [29]. In clinical research, the coexistence of CagA and VacA genes has also been confirmed. Some studies suggest that the combination of CagA-positive and VacA s1m1 genes is more common in patients with gastric cancer, and this combination may be related to an increased risk of gastric cancer [30]. The results of this study showed that among the 127 Hp antibody-positive patients, 67 were infected with Type I Hp, and all had positive CagA antibodies, that is, the positive rate of CagA antibodies in Hp antibody-positive patients was 52.76% (67/127), which is lower than demonstrated in previous research data [27]. This may be related to differences in gene expression, and the fact that the CagA protein in the stomach of infected individuals may not directly stimulate the immune system to produce the corresponding antibodies. Additionally, the disappearance of antibodies after Hp eradication treatment may also contribute to this phenomenon. The 33 patients with positive VacA antibodies were all positive for CagA antibodies, which is consistent with previous research reports [30], further supporting the synergistic role of CagA and VacA in Hp infection, and this may have an impact on the clinical outcomes of Hp infection.

The symptoms of Hp infection are usually non-specific, which makes diagnosis and treatment complex. Most infected individuals may present with no symptoms or only mild digestive discomfort, which are often similar to the presentation of other gastrointestinal diseases [19]. Some infected individuals may not even develop any obvious clinical symptoms, making the identification of infection more difficult [31]. Hp infection can be diagnosed through the UBT, gastroscopy, and histology. The UBT is the most commonly used non-invasive method for detecting Hp infection in the human body, and is recommended as the first choice for current infection and post-eradication follow-up [11,12,13]. Histological examination remains an irreplaceable reliable method for diagnosing Hp infection, as it can not only confirm the presence of Hp infection, but also elucidate the condition of the gastric mucosal tissue [19]. A previous clinical study in Beijing, incorporating Hp-sATT qualitative analysis, suggested that the consistency between the Hp-sATT and ^13^C-UBT was moderate (Kappa = 0.52), and the consistency between the Hp-sATT and SS-Hp was poor (Kappa = 0.30), with Type I Hp infection leading to more obvious gastric diseases [32], but the impact of Hp eradication treatment on serological antibodies was not considered. This study suggested that the consistency between the Hp-sATT and 13C-UBT is poor (Kappa = 0.339), and the consistency between the Hp-sATT and SS-Hp is moderate (Kappa = 0.427). Although no significant correlation was found between the Hp-sATT and the severity of gastric diseases, there is a clear relationship between current Hp infection and gastric diseases in patients with a positive ^13^C-UBT. This finding underscores the pathogenicity of Hp infection. However, with the expansion of indications for Hp eradication therapy, the clinical utility of the Hp-sATT as a diagnostic marker may diminish in contemporary practice.

In addition, we also observed that in patients without a history of Hp eradication, the signal value of anti-urease antibody detection and the DOB value of the ^13^C-UBT were positively correlated. Although there are differences in the consistency of Hp antibody detection and the ^13^C-UBT in diagnosing Hp infection, they may have been a certain synergistic effect in the quantitative results of the testing, and this positive correlation may reflect the correlation of anti-urease antibodies and DOB value with urease, and also support the role of this enzyme in the histopathological process. Specifically, the urease produced by Hp decomposes urea into ammonia and carbon dioxide, creating an alkaline environment that neutralizes stomach acid and allows the bacteria to survive in the acidic gastric milieu [33]. This mechanism not only facilitates the survival and colonization of Hp in the stomach, but also contributes to its pathogenicity by damaging the gastric mucosa and triggering inflammation [34]. The production of anti-urease antibodies is part of the immune response to Hp infection, and the increase in DOB value is directly related to the activity of Hp’s urease; both are related to the presence and activity of Hp infection [2,23]. Notably, anti-urease antibody levels not only demonstrate diagnostic utility for Hp infection, but also serve as an indicator of the host’s adaptive immune recognition of the pathogen. This also suggests that in patients with primary Hp infection, the Hp-sATT and ^13^C-UBT may have complementary value in monitoring the severity of Hp infection and the effectiveness of treatment.

Where the impact of Hp eradication treatment is concerned, we found that in the population without a history of Hp eradication, the consistency between the Hp-sATT, ^13^C-UBT, and SS-Hp improved, with an especially high positive agreement rate between the Hp-sATT and ^13^C-UBT. This suggests that for individuals who have not undergone Hp eradication treatment and are not suitable for invasive procedures such as gastroscopy, the Hp-sATT and ^13^C-UBT still hold certain clinical value in screening for Hp infection. In summary, with the expansion of the screening and treatment range of Hp infection in recent years, the limitations of the traditional Hp-sATT in assessing the status of Hp infection have gradually emerged. That is, antibodies persist in patients post-eradication, and reliance on the Hp-sATT for clinical decision-making carries risks. More emphasis should be placed on more direct and accurate detection methods, such as the UBT and histological examination. This study was a single-center retrospective clinical study, and the above discussions still need to be further clarified by studies with a higher level of evidence.

## 5. Conclusions

Clinically, the patient’s Hp eradication history must be prioritized in the interpretation of Hp-sATT results. In patients without a history of Hp eradication, the consistency between the Hp-sATT and ^13^C-UBT is moderate, and the positive correlation between the signal value of anti-urease antibody detection and the DOB value of the ^13^C-UBT also indicates their application value in patients without a history of eradication. This study is based on data from a single center, and is specific to outpatients in the gastroenterology department. Therefore, the results of positive Hp-sATT and ^13^C-UBT rates should not be extrapolated to broader populations.

## Figures and Tables

**Table 1:** Comparison between Hp-infected and non-infected groups.

		Active Hp Infection (n = 107)	Non-Active Hp Infection (n = 130)	*p*
Gender	Male	56 (52.3)	54 (41.5)	0.097 ^a^
	Female	51 (47.7)	76 (58.5)	
Age (years)		48.18 ± 13.46	45.85 ± 14.20	0.154 ^b^
No upper gastrointestinal symptoms		17 (15.9)	18 (13.8)	0.194 ^a^
Upper gastrointestinal symptoms	Nausea	8 (7.5)	11 (8.5)	0.781 ^a^
	Vomiting	6 (5.6)	1 (0.8)	0.071 ^c^
	Regurgitation	27 (25.2)	33 (25.4)	0.979 ^a^
	Heartburn	14 (13.1)	14 (10.8)	0.583 ^a^
	Belching	18 (16.8)	25 (19.2)	0.632 ^a^
	Abdominal pain	34 (31.8)	38 (29.2)	0.672 ^a^
	Abdominal distension	49 (45.8)	62 (47.7)	0.771 ^a^

Note: Data are presented as mean ± SD, or No. (%). ^a^ Pearson Chi-Square. ^b^ Mann–Whitney U test. ^c^ Yates’ Chi-square test.

**Table 2:** Comparison of Hp-sATT and ^13^C-UBT.

		^13^C-UBT Positive	^13^C-UBT Negative	*p*	Kappa
Hp-sATT for All Patients (n = 237)	Positive	76	51	<0.001	0.339
	Negative	28	82		
Hp-sATT for Patients with Eradication History (n = 46)	Positive	10	20	0.270	−0.142
	Negative	8	8		
Hp-sATT for Patients without Eradication History (n = 191)	Positive	66	31	<0.001	0.467
	Negative	20	74		

**Table 3:** Comparison of Hp-sATT and ^13^C-UBT with SS-Hp.

		SS-Hp Positive	SS-Hp Negative	*p*	Kappa
Hp-sATT for All Patients (n = 127)	Positive	40	33	<0.001	0.427
	Negative	5	49		
Hp-sATT for Patients with Eradication History (n = 20)	Positive	4	8	0.068	0.286
	Negative	0	8		
Hp-sATT for Patients without Eradication History (n = 107)	Positive	36	25	<0.001	0.457
	Negative	5	41		
^13^C-UBT for All Patients (n = 127)	Positive	42	21	<0.001	0.621
	Negative	3	61		
^13^C-UBT for Patients with Eradication History (n = 20)	Positive	4	5	0.013	0.468
	Negative	0	11		
^13^C-UBT for Patients without Eradication History (n = 107)	Positive	38	16	<0.001	0.646
	Negative	3	50		

**Table 4:** Comparison of Hp-sATT and ^13^C-UBT with gastroscopy and/or histopathological diagnosis.

			Gastritis	Erosion	Ulcer	Atr/IM/Dys	*p*
SHp-sATT (n = 237)	Positive (n = 127)	Type I (n = 67)	33 (24.09)	5 (22.72)	6 (54.55)	23 (34.33)	0.266 ^a^
		Type II (n = 60)	35 (25.55)	6 (27.27)	1 (9.09)	18 (26.87)	
		Total	68 (49.64)	11 (50.00)	7 (63.64)	41 (61.19)	0.395 ^a^
	Negative (n = 110)		69 (50.36)	11 (50.00)	4 (36.36)	26 (38.81)	
^13^C-UBT (n = 237)	Positive (n = 104)		47 (34.31)	10 (45.45)	7 (63.64)	40 (59.70)	0.003 ^a^
	Negative (n = 133)		90 (65.69)	12 (54.55)	4 (36.36)	27 (40.30)	

Note: Atr/IM/Dys: atrophy/intestinal metaplasia/dysplasia. ^a^ Pearson Chi-square.

## Data Availability

The raw data supporting the conclusions of this article will be made available by the authors on request.

## References

[1] Li Y., Choi H., Leung K., Jiang F., Graham D.Y., Leung W.K. (2023). Global prevalence of *Helicobacter pylori* infection between 1980 and 2022: A systematic review and meta-analysis. Lancet Gastroenterol. Hepatol..

[2] Yamasaki S., Murata M., Ohta A., Matsumoto Y., Ikezaki H., Furusyo N. (2024). Analyses of the association between *Helicobacter pylori* antibody titre and pathogenicity before and after eradication: Results of the Kyushu and Okinawa population study, a retrospective observational cohort study. BMJ Open.

[3] Fakharian F., Asgari B., Nabavi-Rad A., Sadeghi A., Soleimani N., Yadegar A., Zali M.R. (2022). The interplay between *Helicobacter pylori* and the gut microbiota: An emerging driver influencing the immune system homeostasis and gastric carcinogenesis. Front. Cell. Infect. Microbiol..

[4] Yang Y., Shu X., Xie C. (2022). An Overview of Autophagy in *Helicobacter pylori* Infection and Related Gastric Cancer. Front. Cell. Infect. Microbiol..

[5] Doocey C.M., Finn K., Murphy C., Guinane C.M. (2022). The impact of the human microbiome in tumorigenesis, cancer progression, and biotherapeutic development. BMC Microbiol..

[6] Liu W., Tian J., Hui W., Kong W., Feng Y., Si J., Gao F. (2021). A retrospective study assessing the acceleration effect of type I *Helicobacter pylori* infection on the progress of atrophic gastritis. Sci. Rep..

[7] Yuan L., Zhao J.-B., Zhou Y.-L., Qi Y.-B., Guo Q.-Y., Zhang H.-H., Khan M.N., Lan L., Jia C.-H., Zhang Y.-R. (2020). Type I and type II *Helicobacter pylori* infection status and their impact on gastrin and pepsinogen level in a gastric cancer prevalent area. World J. Gastroenterol..

[8] Chinese Society of Health Management, National Clinical Research Center for Digestive Diseases (Shanghai), Helicobacter pylori Group of Chinese Society of Gastroenterology, Journal of Health Examination and Management, Health Management and Physician Health Insurance Professional, Committee of Chinese Medical Doctor Association (2022). Expert Consensus on Serological Detection of Helicobacter pylori in Physical Examination Populations (2022). J. Health Exam. Manag..

[9] Malfertheiner P., Megraud F., Rokkas T., Gisbert J.P., Liou J.-M., Schulz C., Gasbarrini A., Hunt R.H., Leja M., O’Morain C. (2022). Management of *Helicobacter pylori* infection: The Maastricht VI/Florence consensus report. Gut.

[10] Chinese Medical Association Health Management Branch, Editorial Committee of Chinese Journal of Health Management, *Helicobacter pylori* Group of Chinese Society of Gastroenterology (2021). Expert consensus on ^13^C urea breath test in health check-ups. Chin. J. Health Manag..

[11] Shah S.C., Iyer P.G., Moss S.F. (2021). AGA Clinical Practice Update on the Management of Refractory *Helicobacter pylori* Infection: Expert Review. Gastroenterology.

[12] Sugano K., Tack J., Kuipers E.J., Graham D.Y., El-Omar E.M., Miura S., Haruma K., Asaka M., Uemura N., Malfertheiner P. (2015). Kyoto global consensus report on *Helicobacter pylori* gastritis. Gut.

[13] Liya Z., *Helicobacter pylori* Study Group, Chinese Society of Gastroenterology, Chinese Medical Association (2022). Sixth National Consensus Report on the Management of *Helicobacter pylori* Infection (Non-Eradication Treatment Section). Chin. J. Dig..

[14] Katelaris P., Hunt R., Bazzoli F., Cohen H., Fock K.M., Gemilyan M., Malfertheiner P., Mégraud F., Piscoya A., Quach D. (2023). *Helicobacter pylori* World Gastroenterology Organization Global Guideline. J. Clin. Gastroenterol..

[15] Bosch D.E., Krumm N., Wener M.H., Yeh M.M., Truong C.D., Reddi D.M., Liu Y., Swanson P.E., Schmidt R.A., Bryan A. (2020). Serology Is More Sensitive Than Urea Breath Test or Stool Antigen for the Initial Diagnosis of *Helicobacter pylori* Gastritis When Compared With Histopathology. Am. J. Clin. Pathol..

[16] Butt J., Blot W.J., Shrubsole M.J., Varga M.G., Hendrix L.H., Crankshaw S., Waterboer T., Pawlita M., Epplein M. (2020). Performance of multiplex serology in discriminating active vs past *Helicobacter pylori* infection in a primarily African American population in the southeastern United States. Helicobacter.

[17] Su J.Y., Liu C.T., Wang T.S., Li W.K., Yang Y., Wu S.S., Li P., Wu J. (2022). Correlation between *Helicobacter pylori* antibody typing detection in serum and gastric mucosal lesions. J. Capital. Med. Univ..

[18] Wang X., Zheng S.Q., Fu Q.H., He X.D. (2023). Advances in the relationship between *Helicobacter pylori* and its typing and gastrointestinal diseases. Int. J. Dig. Dis..

[19] Malfertheiner P., Camargo M.C., El-Omar E., Liou J.-M., Peek R., Schulz C., Smith S.I., Suerbaum S. (2023). *Helicobacter pylori* infection. Nat. Rev. Dis. Primers.

[20] Ren S., Cai P., Liu Y., Wang T., Zhang Y., Li Q., Gu Y., Wei L., Yan C., Jin G. (2022). Prevalence of infection in China: A systematic review and meta-analysis. J. Gastroenterol. Hepatol..

[21] Li Z.S., Yang Y.S., Lv N.H., Zhou L.Y., Ding S.Z., National Clinical Research Center for Digestive Diseases (Shanghai), National Early Cancer Prevention and Control Center Alliance, *Helicobacter pylori* and Peptic Ulcer Group of Chinese Society of Gastroenterology, *Helicobacter pylori* Chinese Association of *Helicobacter pylori* Study (2021). Expert consensus on the prevention and management of *Helicobacter pylori* infection in Chinese residents (2021). Chin. J. Dig..

[22] Chinese Society of Pediatrics, Gastroenterology Group, National Children’s Medical Center, Gastroenterology Alliance, Editorial Board of Chinese Journal of Pediatrics (2023). Expert consensus on the diagnosis and treatment of *Helicobacter* pylori infection in Chinese children (2022). Chin. J. Pediatr..

[23] National Clinical Research Center for Digestive Diseases (Shanghai), Chinese Society of Health Management, Chinese Society of Nuclear Medicine (2020). Expert consensus on the clinical application of *Helicobacter pylori*-urea breath test (2020). Chin. J. Dig..

[24] Ichihara A., Ojima H., Gotoh K., Matsushita O., Take S., Okada H., Watanabe A., Yokota K. (2021). Serodiagnosis and Bacterial Genome of *Helicobacter pylori* Infection. Toxins.

[25] Knorr J., Ricci V., Hatakeyama M., Backert S. (2019). Classification of *Helicobacter pylori* Virulence Factors: Is CagA a Toxin or Not?. Trends Microbiol..

[26] Ansari S., Yamaoka Y. (2020). Role of vacuolating cytotoxin A in *Helicobacter pylori* infection and its impact on gastric pathogenesis. Expert Rev. Anti-Infective Ther..

[27] Zhu X., Ma C., Sa R., Wang Y., Zhu C., Zhao Y., Luo J., Liu X. (2024). CagA 3′ region polymorphism of *Helicobacter pylori* and its association with chronic gastritis in the Chinese population. J. Med. Microbiol..

[28] Ni H.K., Liao L.M., Huang R.L., Zhou W. (2020). The relationship between gastric cancer and *Helicobacter pylori* cytotoxin-related gene A genotypes. Cell. Mol. Biol..

[29] Sheikh A.F., Yadyad M.J., Goodarzi H., Hashemi S.J., Aslani S., Assarzadegan M.-A., Ranjbar R. (2018). CagA and vacA allelic combination of *Helicobacter pylori* in gastroduodenal disorders. Microb. Pathog..

[30] Xue Z., Li W., Ding H., Pei F., Zhang J., Gong Y., Fan R., Wang F., Wang Y., Chen Q. (2024). Virulence gene polymorphisms in Shandong *Helicobacter pylori* strains and their relevance to gastric cancer. PLoS ONE.

[31] Yu T., Lu T., Deng W., Yao D., He C., Luo P., Song J. (2023). Microbiome and function alterations in the gastric mucosa of asymptomatic patients with *Helicobacter pylori* infection. Helicobacter.

[32] Zhang M., Zhao Z., Li X., Jin Y., Ren Y., Jiang B., Jiang X., Zhao X. (2020). Correlation between *Helicobacter pylori* antibody typing and gastric diseases. J. Mark. Immunoanal. Clin..

[33] Yang H., Huang X., Zhang X., Zhang X., Xu X., She F., Wen Y. (2022). AI-2 Induces Urease Expression Through Downregulation of Orphan Response Regulator HP1021 in *Helicobacter pylori*. Front. Med..

[34] Graham D.Y., Miftahussurur M. (2018). *Helicobacter pylori* urease for diagnosis of *Helicobacter pylori* infection: A mini review. J. Adv. Res..

